# Predicting the spatio-temporal distribution of the invasive alien plant *Andropogon virginicus*, in the South Korean peninsula considering long-distance dispersal capacities

**DOI:** 10.1371/journal.pone.0291365

**Published:** 2023-11-14

**Authors:** Jeong-Soo Park, Hyohyemi Lee

**Affiliations:** Division of Climate Change Research, National Institute of Ecology, Seocheon, Korea; University of Delhi Department of Environmental Studies, INDIA

## Abstract

The spread of invasive alien species is a major threat to biodiversity. Estimating the long-distance dispersal capacity of invasive alien plants is vital for understanding their population dynamics and community composition. We predicted the spatial-temporal distribution of the alien plant *Andropogon virginicus*, in the Korean peninsula under climate change scenario using Random Forest (RF) and Cellular Automaton (CA) methods. Land use, barriers to dispersal, long-distance dispersal frequency, and maximum long-distance dispersal range were considered in our analysis. Our results showed that, among the five selected environmental variables, annual mean temperature and Human Foot-Printing (HFP) were positively associated with the occurrence probability of *A*. *virginicus*. This suggests that *A*. *virginicus* is likely to spread to the disturbed northern part of the Korean Peninsula due to climate change and habitat preference. When comparing modeling results for dispersal to field survey data, the modeling raster sets drawn from the long-distance dispersal frequency of 0.05 and maximum long-distance dispersal distance of 30 km y^-1^ had the most similar spatial expansion among the six long-distance dispersal parameter sets. The dispersal directions were associated with the landscape. Specifically, seeds dispersed by wind (anemochorous seeds) could propagate into open landscapes more easily than in forests. Regarding *A*. *virginicus* management, this grass can quickly invade bare ground with their wind-dispersed seeds, therefore habitat destruction, such as excessive logging and weeding, should be restrained.

## Introduction

The spread of invasive alien species has become one of the main threats to biodiversity, in addition to habitat degradation and climate change, etc. [1, 2]. Invasive alien plants can rapidly encroach on the habitats of native endangered species through the dispersal of seeds and separated propagules. In particular, long-distance dispersal (LDD) can contribute to surprisingly fast invasion rates in many species [3]. Hampe [4] studied the long-distance dispersal capabilities of seeds in past and ongoing expansions, reporting that seed dispersal and colonization processes can be altered by rapid climate change. Another study also reported that long-distance dispersal ability is important for estimating the population dynamics and community composition of invasive alien plants under climate change [5].

*Andropogon virginicus* (Poaceae) is a perennial C_4_ grass species. This species is native to the southeast United States and as far north as the Great Lakes, and had been introduced in several countries including Australia, New Zealand, France, Japan, and South Korea [6, 7]. The recent rapid expansion of *A*. *virginicus* has raised concerns regarding ecosystem management and conservation efforts on the South Korean Peninsula. The stands of this plant can be dense and highly competitive because of allelopathic substances, which can cause a biodiversity decline in the invaded habitats [8]. *Andropogon virginicus* grows in various soils and in open habitats such as abandoned croplands, roadsides, pastures, and open woodlands. It is a prolific producer with approximately 500 spikelets per flowering culm, which are dispersed by wind from early October to mid-December [8]. Furthermore, this plant can promote fires during the dry winter season due to dead aboveground biomass and can recover quickly after the fires from the wind-dispersed seeds [7].

We predicted the spatial-temporal distribution of this plant under the climate change scenario. The traditional species distribution model (habitat suitability model) can only predict the potential distribution areas of species regardless of species-specific dispersal ability, which determines the possibility of its migration into the new habitat [9–12]. Estimating species’ dispersal capacities and habitat constraints can provide more realistic forecasts of their future distribution [13]. In this study, we focused on the LDD frequency and maximum dispersal range because this information is critical for determining the likelihood of wind-dispersed species migrating into a new suitable habitat. Several studies have measured the dispersal distances of plants in the field [14, 15] and developed mathematical models to estimate dispersal distances [16–18]. However, it is difficult to estimate long-distance dispersal ability because it depends on various factors such as plant traits (e.g. seed mass, morphology, height of seed release), environmental conditions (e.g. wind speed, turbulence, landscape), and dispersal vectors (e.g. wind, water, animal) [19–22]. We analyzed the long-distance dispersal capacities of *A*. *virginicus* by comparing modeling results for dispersal to field survey data, and then we predicted its distribution under the climate change scenario.

This study aimed to (1) predict the potential distribution of *A*. *virginicus* under climate change scenario, (2) estimate the long-distance dispersal capacity of this plant, and (3) identify the environmental variables critical for the distribution of this alien plant.

## Methods

### Species occurrence data and study area

We obtained 7,550 occurrence points of *A*. *virginicus* in North America from the Global Biodiversity Information Facility database (GBIF, https://www.gbif.org/species/2706080) [23]. We excluded points that have high coordinate uncertainty (> 100 m) and are close to each other (< 30 km) considering our hypothesis of long-distance dispersal capacities. Finally, 200 points were selected as occurrence points in North America for the habitat suitability model. We randomly selected pseudo-absence points in North America with distances over 110 km (approximately 1° latitude/longitude) from other points to avoid similar environmental conditions and to decrease spatial autocorrelation of residuals according to Moran’s I statistics (-0.003, *p*-value = 0.560) [24]. The number of pseudo-absences was determined based on the model performance. Model performance was evaluated based on four different criteria such as continuous Boyce index (CBI), the area under the receiver operating characteristic curve (AUC), kappa statistic, and true skill statistic (TSS) (S1 Table, S1 Fig). We considered CBI to be the most important criterion among them because it is designed specifically for testing models derived from presence-only data [25]. Finally, our dataset comprised 1,000 points (200 presence points and 800 pseudo-absence points). We also collected 23 populations of *A*. *virginicus* in our study area, the Jeolla and Chungcheong provinces of South Korea, from the Convention on Biological Diversity-Clearing House Mechanism Korea website [26] and a field survey. The field survey was conducted in 2021 and 2022, we recorded the Global Positioning System (GPS) coordinates of the population of *A*. *virginicus* and measured its area. The largest area of the population was 43 ha and the smallest one was 9 m^2^. *A*. *virginicus* was initially detected in Gunsan (36° 00′ 54″, 126° 45′ 36″) and Hwasun (34° 55′ 23″, 126° 53′ 22″) in 2009 and spread to other areas over the last thirteen years [24].

**Fig 1:**
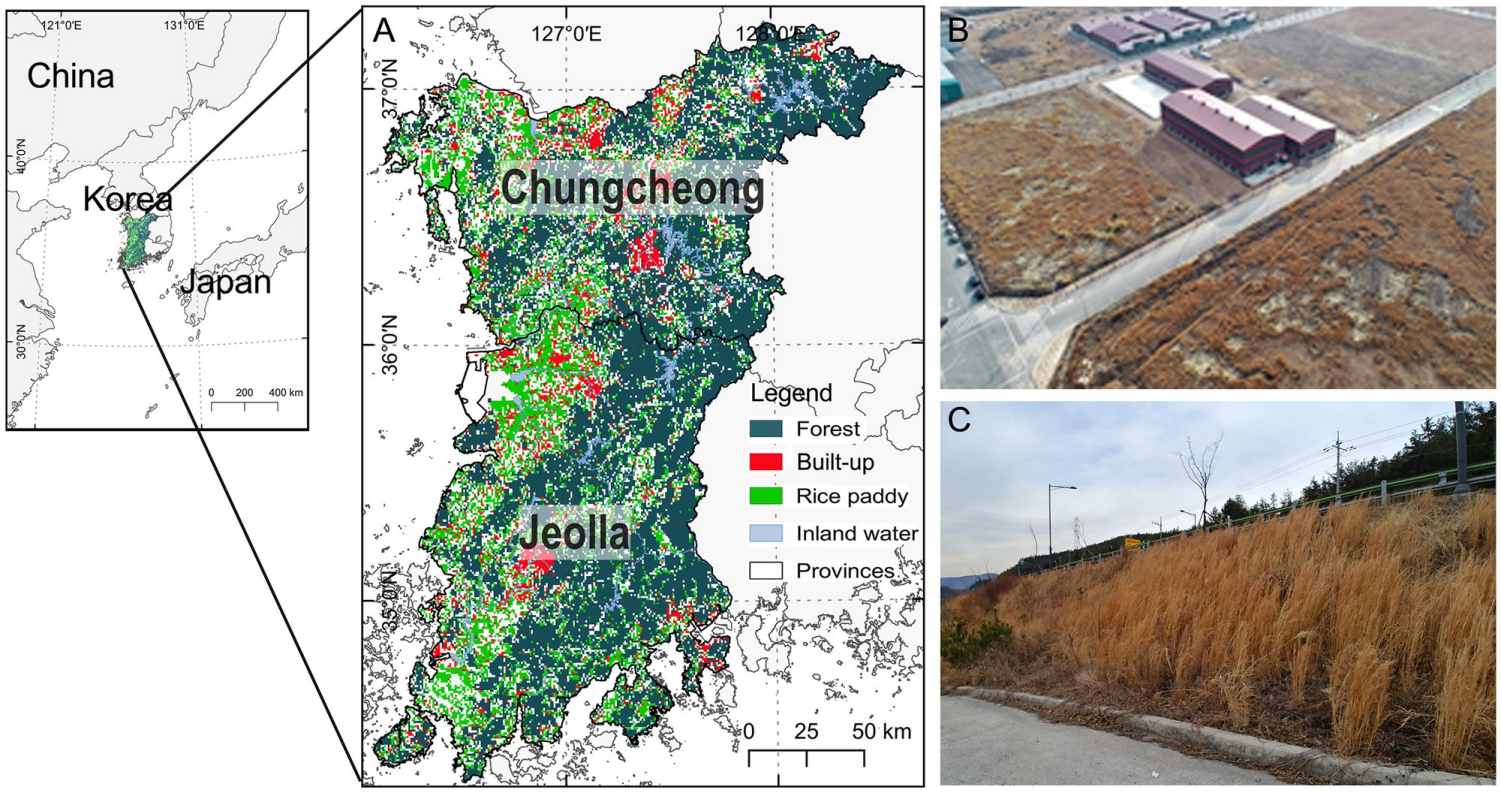
(A) Location of the study area (Chungcheong and Jeolla provinces) in South Korea showing the four land use classes (forest, built-up area, rice-paddy, and inland water). (B) The largest population of *A*. *virginicus* is in South Korea (43 ha). (C) The population of *A*. *virginicus* is located on the roadside. The base map is an open-source shapefile from the National Geographic Information Institute of Korea [30].

The total area of our study site is 36,038 km^2^ located in Korean Peninsula (Fig 1). The annual mean temperature is 11.8°C and the mean precipitation is 1,301 mm over the last 30 years [27]. The soil parent material consists of mainly acidic and metamorphic rocks [28]. The land cover map was downloaded from the environmental geographic information service (EGIS, https://egis.me.go.kr) [29]. The three main land-cover classes in our study area were forest (46%), farmland (39%), and residential (11%). We excluded islands from further analyses because of the lower accessibility and possibility of seed dispersal compared to inland regions. We used QGIS 3.22.5 software to visualize the site maps and the spatial distribution of *A*. *virginicus* based on the open-source shapefile from the National Geographic Information Institute of Korea (NGII, http://www.ngii.go.kr) [30].

### Habitat suitability modeling

The suitable habitat of *A*. *virginicus* in North America was estimated using a random forest (RF) model using the “randomForest” package in R 4.1.2 [31]. The set of initial explanatory variables were categorized into three groups based on their information properties and data sources: 19 bioclimatic variables from the WorldClim database (https://www.worldclim.org) [32], the Global Human Foot-Printing (HFP) as an anthropogenic variable from the SocioEconomic Data and Applications Center (SEDAC, http://sedac.ciesin.columbia.edu/), which is derived from nine global data layers (population density, population settlements, built-up areas, nighttime lights, land use/land cover, coastlines, roads, railroads, and navigable rivers). HPF expresses as a percentage the relative human influence in each terrestrial biome [33], and elevation as the geographical variable from the National Geographic Information Institute of Korea [30]. To avoid multicollinearity among variables and model over-fit in the model, we calculated the correlation between pairs of variables and the variance inflation factor (VIF) (S2 Fig). The pair of variables with the highest correlation value (>0.8) was selected and the variable with a higher VIF was excluded from the pair. This procedure was repeated until the VIF value was lower than 4 for all selected variables [34]. After backward stepwise selection, the final variables retained in the model were: three bioclimatic variables (bio01: annual mean temperature; bio12: annual precipitation; bio14: precipitation of the driest month), Human Foot-Printing (HFP), and elevation.

All digital raster maps of North America used identical resolution (1 × 1 km cell size) and geographic coordinate systems (WGS84, EPGS4326). The extracted data (n = 1000) were randomly divided into training and testing datasets in a ratio of 7:3. Model accuracy was evaluated using four different criteria such as continuous Boyce index (CBI), the area under the receiver operating characteristic curve (AUC), kappa statistic, and true skill statistic (TSS) using the “enmSdmX” package [35] and “PresenceAbsence” package [36] in R. To estimate the relationships between the occurrences of *A*. *virginicus* and the selected environmental variables, we calculated the reduction in node impurity and partial dependence of occurrence probability using the “randomForest” package in R [31].

The developed model was then used to project habitat suitability in the study area under the climate change scenario. The current and future climate raster datasets for our study areas were downloaded from the WorldClim database (version 2.1) (http://worldclim.org/data/) with a spatial resolution of 30 seconds [32]. These climate data were based on the shared socioeconomic pathway (SSP) of the Model of Interdisciplinary Research on Climate (MIROC6). This climate change scenario was selected because it focused on the climatic mean states and internal climate variability in the East Asian monsoon region [37]. We applied the SSP3-7.0, which is in the upper-middle part of the full range of scenario, compared to other optimistic and catastrophic scenarios. SSP3-7.0 assumes that temperatures will reach above 3°C than the pre-industrial period, and substantial land use change will occur [38, 39]. Two periods were considered for this analysis, 2041 (2041–2060) and 2061 (2061–2080). We downscaled the suitable habitat maps (final resolution: 200 × 200 m) using the ordinary kriging method. Kriging interpolation was performed using the package “gstat” in R [40].

### Dispersal simulation

**Fig 2:**
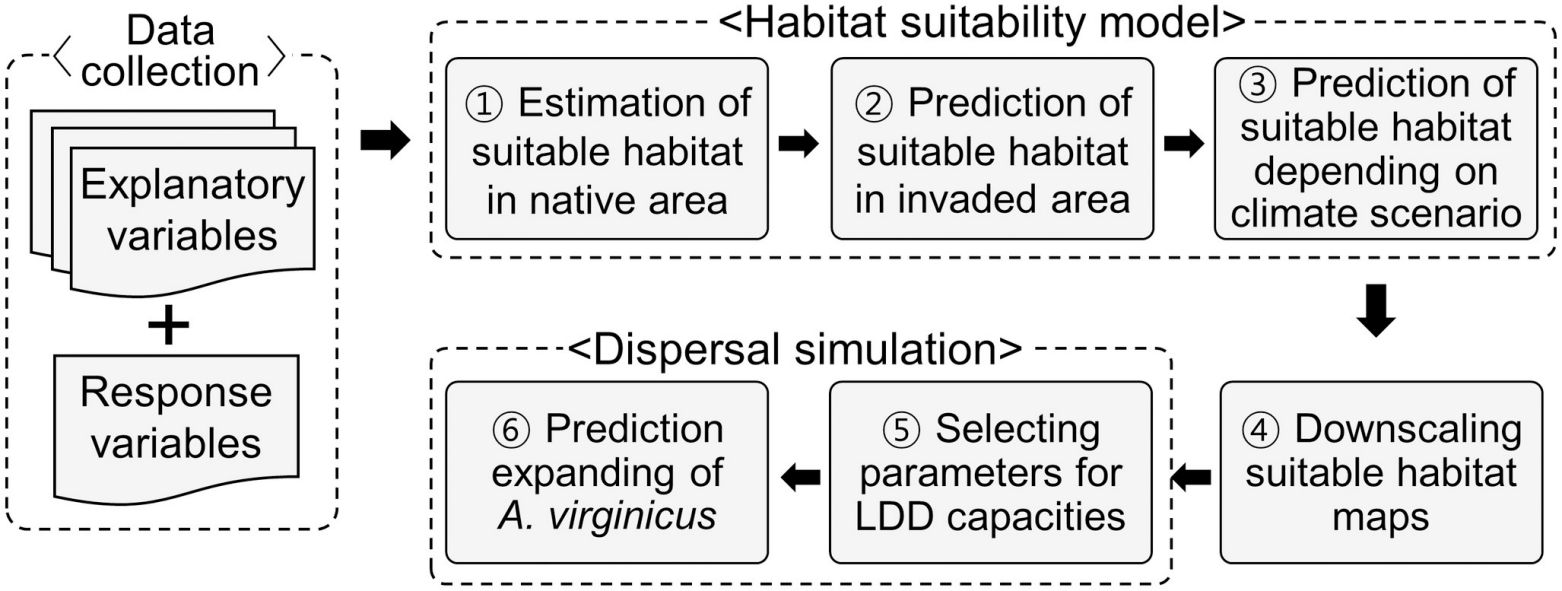
Flow-chart of a dispersal simulation for *A*. *virginicus*.

“MigClim” and “SDMTools” are built-in packages in the R software designed to implement species-specific dispersal constraints into the projections of species habitat suitability models (Fig 2) [41, 42]. It is a cellular automaton model linked to habitat suitability maps under climate change scenario and landscape fragmentation [43]. Landscape fragmentation represents the dispersal barrier (forest) and unsuitable habitats (build-up area, rice-paddy, and water bodies). This model requires the following inputs: 1) an initial distribution map of the species based on field survey data; 2) a series of maps picturing suitable habitats under climate change scenario; 3) dispersal parameters such as dispersal kernel, propagule production potential, and LDD; and 4) landscape fragmentation maps (i.e., dispersal barriers and unsuitable habitats). The seed dispersal behavior of the species was determined according to the seed type. The initial distributions of *A*. *virginicus* were assumed to be spread across a radius of 5 km from the detected points because of the dispersal efficiency of its plumes and anthropogenic effects [21]. In detail, although the dispersal information of *A*. *virginicus* has not been reported concretely, it is well known that its seeds are mainly dispersed by wind or wheel of vehicles because of the low seed weight and the structure of pappus [6].

Long-distance dispersal (LDD) can be defined depending on the relative frequency of extreme dispersal events (e.g., 0.1% of all seeds) or the specified threshold distance (e.g., 10 km) [44]. We considered three LDD frequencies (0.1%, 0.05%, and 0.01%) and two maximum LDD ranges (10 km y^-1^ and 30 km y^-1^), and applied six different LDD parameter sets (F0.1_10 km, F0.05_10 km, F0.01_10 km, F0.1_30 km, F0.05_30 km, F0.01_30 km). we compared the results of dispersal models under six different LDD parameter sets with the field survey data conducted in 2009 and 2022. To determine how well each LDD parameter predicted the spread of *A*. *virginicus*, the results from the model were compared with the field survey data using the modified Jaccard similarity index derived from the pixels of the modeled dispersal against the actual dispersal of *A*. *virginicus* [45, 46].

To decide the initial distribution area, we assumed that the populations with distances less than 10 km from each other were the same populations based on the distance dispersal capacities of the species with efficient plumes and anthropogenic effects [21]. Finally, ten regions were designated as initial distribution areas in 2021 (Fig 6). We defined the short-distance dispersal (SDD) as the potential dispersal frequency of 99% of all seeds and the maximum dispersal distance was assumed less than 600 m [21]. The negative exponential kernel shape (*D*_(*d*:*λ*)_ = *e*^(−*λd*)^, *λ* = 0.005, *d*: distance (*m*) from the initial distribution area), one of the most common seed dispersal kernels [47], was applied to reflect the higher SDD probability than LDD probability in the model. We assumed that this plant is restricted to build-up area, rice-paddy, and water bodies and is unable to disperse through forested areas (Table 1) [7]. All of the dispersal simulations were replicated 10 times to estimate the level of confidence of our model.

**Table 1:** Parameters used in the MigClim model for the *A*. *virginicus* dispersal estimation.

Parameter	Value	Reference
Dispersal vector	Wind or anthropogenic effects (e.g., human, vehicles)	[6, 21]
Maturity age	1 year	[6–8]
Max. SDD	≤ 600 m	[21]
Min. LDD	> 600 m	
Max. LDD*	≤10 km, ≤30 km	[21, 50]
LDD frequency**	0.1%, 0.05%, 0.01%	
Dispersal event frequency	1 year	[6–8]
Barriers	Forest	[6]
Unsuitable habitats	Build-up area, rice paddy, water bodies	

* LDD events were randomly generated within a distance range of min. LDD (600 m) to max. LDD (10 km or 30 km).

** The success of LDD events, with 0.1, 0.05, or 0.01 events out of 100 being successful. SDD: short-distance dispersal, LDD: long-distance dispersal

## Results

**Fig 3:**
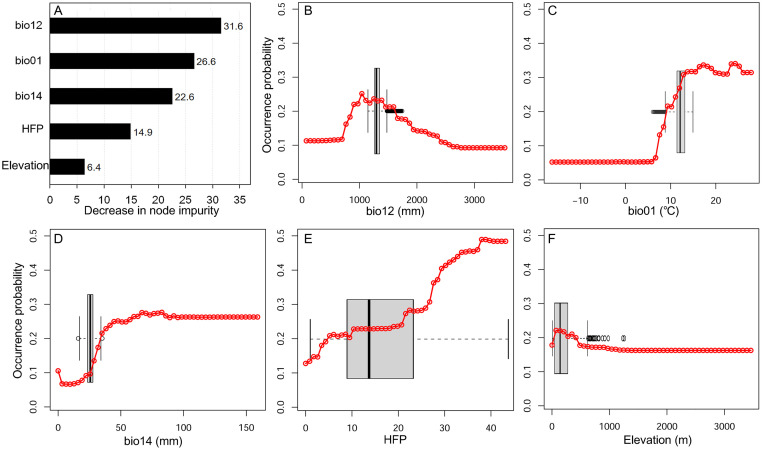
(A) The relative importance of independent variables calculated using node impurity in random forest (bar plot) and the partial dependence of occurrence probability of *A*. *virginicus* against the changes in each environmental variable in North America (red lines). (B) bio12: annual precipitation, (C) Bio01: annual mean temperature, (D) bio14: precipitation of the driest month, (E) HFP: human foot-printing, (F) elevation. The boxplots indicate the summary of each variable in the Jeolla and Chungcheong provinces of the Korean Peninsula (introduced habitat).

On comparing the relative importance of the five predictor variables, we found that annual precipitation (bio12) was the most influential predictor (Fig 3). The occurrence probability of *A*. *virginicus* in North America increased at points with annual precipitation of 800 mm and decreased steadily at the points with 1000 mm. The average annual precipitation in introduced habitats of Korean Peninsula was 1301±74 mm. The occurrence probability of *A*. *virginicus* in North America remarkably increased at points with an annual mean temperature of 10°C or higher, and the annual mean temperature (bio01) in our study site was 11.8±1.2°C. The occurrence probability increased sharply until 50 mm precipitation of the driest month in North America. In the case of our site, the precipitation of the driest month was 28.8±3.1 mm. HFP was positively associated with the occurrence probability of *A*. *virginicus*.

Our random forest model showed good performance based on the CBI (0.971), AUC (0.992), and Kappa statistics (0.912) in North America and CBI (0.952), AUC (0.956), and Kappa statistics (0.838) in South Korea (S1 Table). Using the trained parameters of random forest in the native habitats, we predicted the suitable habitats of *A*. *virginicus* in the introduced regions of the Korean Peninsula (S3 Fig). The highly suitable areas were mainly distributed in western Jeolla Province in 2021, and the low suitable habitats were mainly distributed in high elevation areas. The suitable habitats were predicted to expand toward the southern areas of Jeolla Province, because of the enhanced precipitation effect and decreased temperature effect, in 2060.

**Fig 4:**
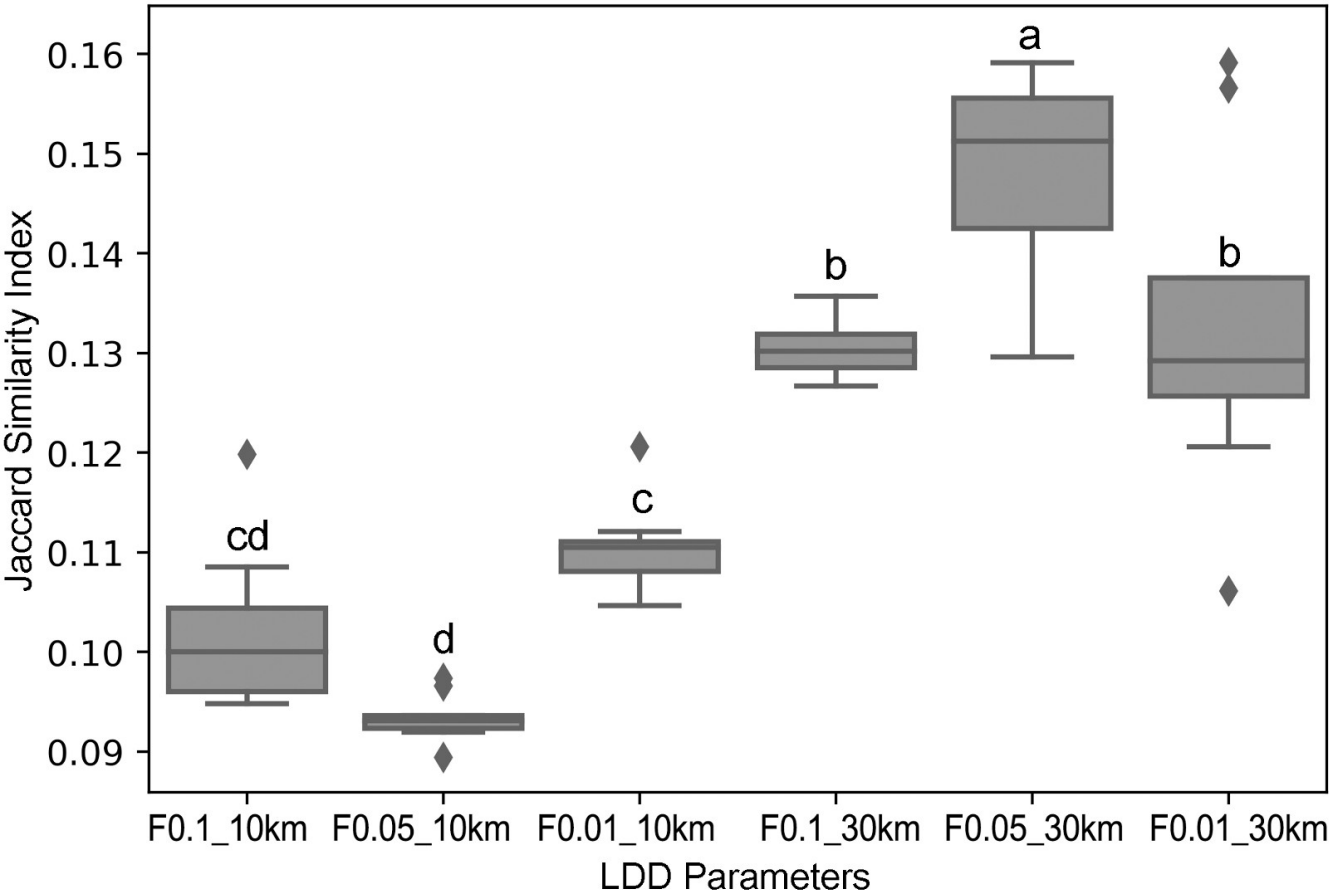
Comparisons of Jaccard similarity index depending on the six LDD parameters. Jaccard Similarity Index represents the similarity between the raster sets of our model and detected regions from the field survey. Values closer to 1 indicate high similarity between the data sets. Meaningful differences of Jaccard similarity index when applied six LDD parameter sets are marked with the different letters (a, b, c, d) based on the results from an analysis of variance (Tukey’s HSD).

Because we did not have specific information related to the LDD capacities of *A*. *virginicus*, the effect of LDD frequency (F0.1, F0.05, and F0.01) and maximum LDD range (10 km y^-1^ and 30 km y^-1^) were modeled, and then compared with the field survey data to select reasonable dispersal parameters (S4 Fig). The modeling raster sets drawn from the long-distance dispersal frequency of 0.05% and maximum long-distance dispersal distance of 30 km y^-1^ had the most similar spatial expansion among the six long-distance dispersal parameter sets (Fig 4). Our model showed that it could not disperse beyond 30 km under the maximum LDD range of 10 km y^-1^. Under the 30 km y^-1^ maximum LDD range, our model projected that this plant could disperse beyond 60 km at an LDD frequency of 0.1% or 0.05%.

**Fig 5:**
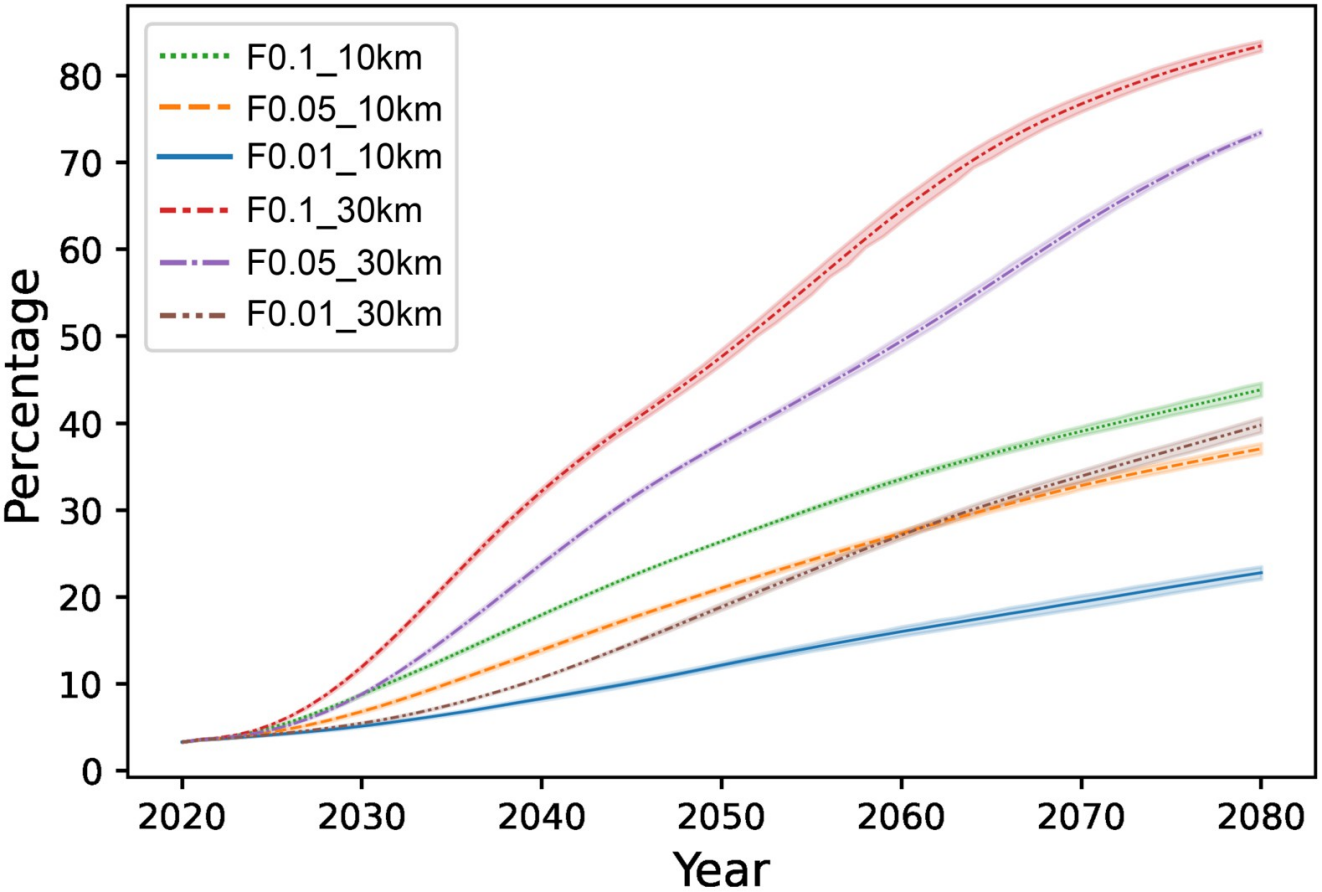
The percentage change of colonized cell in suitable habitats over 60 years based on the three LDD frequencies (F0.1, F0.05, and F0.01) and two maximum LDD ranges (10 km and 30 km). Rich lines represent averages and vague areas display the 95% confidence interval (*n* = 10).

**Fig 6:**
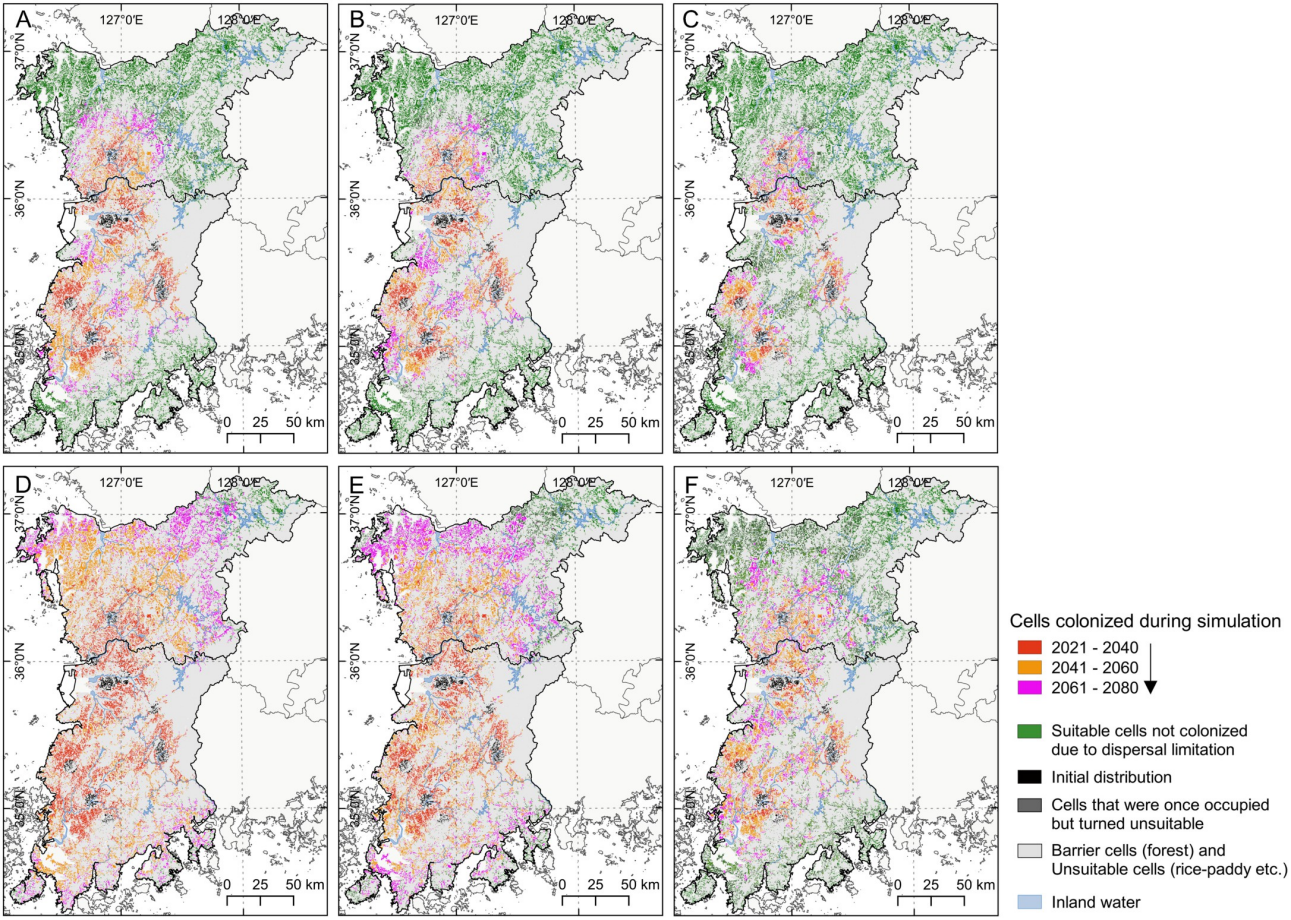
Estimated expansion of *A*. *virginicus* over 60 years based on six LDD parameter sets. [(A) F0.1_10km, (B) F0.05_10km, (C) F0.01_10km, (D) F0.1_30km, (E) F0.05_30km, and (F) F0.01_30km]. The base map is an open source shapefile from the National Geographic Information Institute of Korea [29].

The difference between projections obtained using the six different dispersal parameters was not constant, and their distribution ranges expanded to areas where the climate was suitable (Fig 5). *A*. *virginicus* will expand to 83.4±0.8% of suitable habitats over 60 years under an LDD frequency of 0.1 and a maximum LDD range of 30 km y^-1^. Meanwhile, under an LDD frequency of 0.01 and a maximum LDD range of 10 km y^-1^, this plant occupied 22.7±0.9% of suitable habitats. The expansion rates were remarkably different depending on LDD parameters (Fig 6).

## Discussion

Seed dispersal is an essential process in plants for population dynamics, adaptive evolution, and species persistence [48]. For a more accurate prediction of the changes in *A*. *virginicus* distribution in the future, we applied the MigClim modeling method. This method couples a species distribution model, which predicts the habitat suitability of species using the random forest method, with a cellular automaton model that simulates the dispersal and extinction of species in raster maps [43].

The suitable habitat maps for *A*. *virginicus* indicated a widespread potential for expansion across the southwest Korean Peninsula, especially in open landscape areas. The annual mean temperature and HFP were positively associated with the occurrence probability of *A*. *virginicus*, which suggests that there is an increased risk of *A*. *virginicus* invading the northern areas under climate change. Specifically, the habitats disturbed by human activities, such as abandoned areas, roadsides, deforested areas, and riverbanks, are more susceptible to *A*. *virginicus* invasion within the next 40 years, considering their habitat preferences.

Many studies have measured or estimated the LDD of plants [5, 21, 22], but verification of modeling results with field data is sparse. We compared a set of field survey data with the dispersal modeling results to address the question of LDD capacity. When we compared the modeling results with field survey datasets using the Jaccard similarity index, the modeling raster sets drawn from an LDD frequency of 0.05 and a maximum LDD range of 30 km y^-1^ had the most similar spatial expansion among the six LDD parameter sets. This result indicates that these LDD parameters are the most realistic parameters to predict the dispersal of this grass. A previous study reported that the rate of spread of 16 invasive plant species ranged from 1.4 km y^-1^ to 167 km y^-1^ [49]. For example, the rate of linear spread of the wind dispersed plant species *Epilobium ciliatum* is 9.1 km y^-1^ in the United Kingdom [50].

Diaspore and plant morphological traits, such as seed mass, size, shape, and plant height, are important factors in determining the dispersal distance by wind [20, 51]. *A*. *virginicus* has a low seed weight (> 0.1 mg), and a 2-year-old plant averages 1 m in height. These traits may help in the LDD of its seeds. Heydel et al. [22] reported a significant positive association between seed dispersal capacity and plant traits, such as seed terminal velocity during the fall and height of seed release above the vegetation cover. The seeds of *A*. *virginicus* has a low falling velocity because of the pappus and low seed mass. Furthermore, it can grow taller than other herbal species in open grasslands, which can facilitate the long-distance dispersal of its seeds via strong winds and turbulence.

The direction of dispersal was similar between the modeling results and points detected during the field survey, that is, toward the south-east direction from the northern point and the northwest direction from the southern point. The dispersal directions of this plant were associated with the landscape because seeds dispersed by wind (anemochory) can propagate more easily in open landscapes than in forest landscapes. Williamson et al. [52] also reported that landscape structure and human activity could influence the rate of spread. Additionally, since this C_4_ grass is a shade-intolerant and early seral species [6, 8], the possibility of it invading forests and grasslands is low. This plant is found in a wide variety of open habitats, from roadsides to pasturelands and abandoned croplands [7, 23]. In terms of soil requirements, it can grow on diverse soil textures and low-fertility soils [6]. Furthermore, human activities, such as the movement of vehicles or trains and soil exposure, also facilitate the spread and establishment of invasive alien plants [52]. Our study also demonstrated a positive association between the occurrence of this plant and HFP. We can assume that densely connected roads and populated regions in the Korean peninsula may accelerate the dispersal of this plant.

Our model includes the uncertainties defined by several specific parameters, such as short and long dispersal distance, barriers to dispersal, landscape fragmentation, and increase in reproductive potential over time. Elaborate field experiments should be conducted to figure out the dispersal frequencies and ranges of its seed. Furthermore, several factors that might affect the spread of this plant remain unknown. These include the influence of competition with other species, other dispersal vectors such as animals and water, and habitat changes due to human activities.

We expect that this modeling method can provide more detailed information for the management of invasive alien plants than the traditional habitat suitability model. Our findings indicate that there is a high potential for *A*. *virginicus* to spread into all non-forest ecosystems over the next 60 years. Based on our model, priority areas for monitoring and eradication can be identified for the effective management of this invasive alien plant.

## Supporting information

S1 Fig Predicted versus expected (P/E) curve and continuous Boyce index (CBI) based on the spearman rank analysis for suitable habitat map evaluation depending on the number of pseudo-absences (PS). The upper boxplots represent native habitat (Nat.) and the lower boxplots represent introduced habitat (Int.).
(DOCX)

S2 Fig Spearman correlation matrix for environmental variables in North America. (DOCX)

S3 Fig Maps of the suitable habitat for *A*. *virginicus*. (A) The suitable habitat across its native distribution in North America in 2020. Predicted habitat suitability in the introduced regions in 2021 (B), 2041 (C), and 2061 (D).
(DOCX)

S4 Fig Estimated spatial distribution of *A*. *virginicus* from 2009 to 2021 under six LDD parameter sets. [(A) F0.1_10km, (B) F0.05_10km, (C) F0.01_10km, (D) F0.1_30km, (E) F0.05_30km, (F) F0.01_30km)]. Two blue dots indicate the regions detected in 2009 and red dots indicate the regions detected in 2021 and 2022.
(DOCX)

S1 Table Model validation statistics (CBI, AUC, Kappa, and TSS) for habitat suitability models depending on the number of pseudo-absence points. CBI: continuous Boyce index, AUC: area under the receiver operating characteristic curve, Kappa statistic, TSS: true skill statistic.
(DOCX)
