# Supplementary material for: Predicting the spatio-temporal distribution of the invasive alien plant Andropogon virginicus, in the South Korean peninsula considering long-distance dispersal capacities
Source: PLoS One. 2023 Nov 14;18(11):e0291365. doi: 10.1371/journal.pone.0291365 (PMC10645320; doi:10.1371/journal.pone.0291365)
Supplement: S1 Fig — The upper boxplots represent native habitat (Nat.) and the lower boxplots represent introduced habitat (Int.). (DOCX) [file pone.0291365.s001.docx]

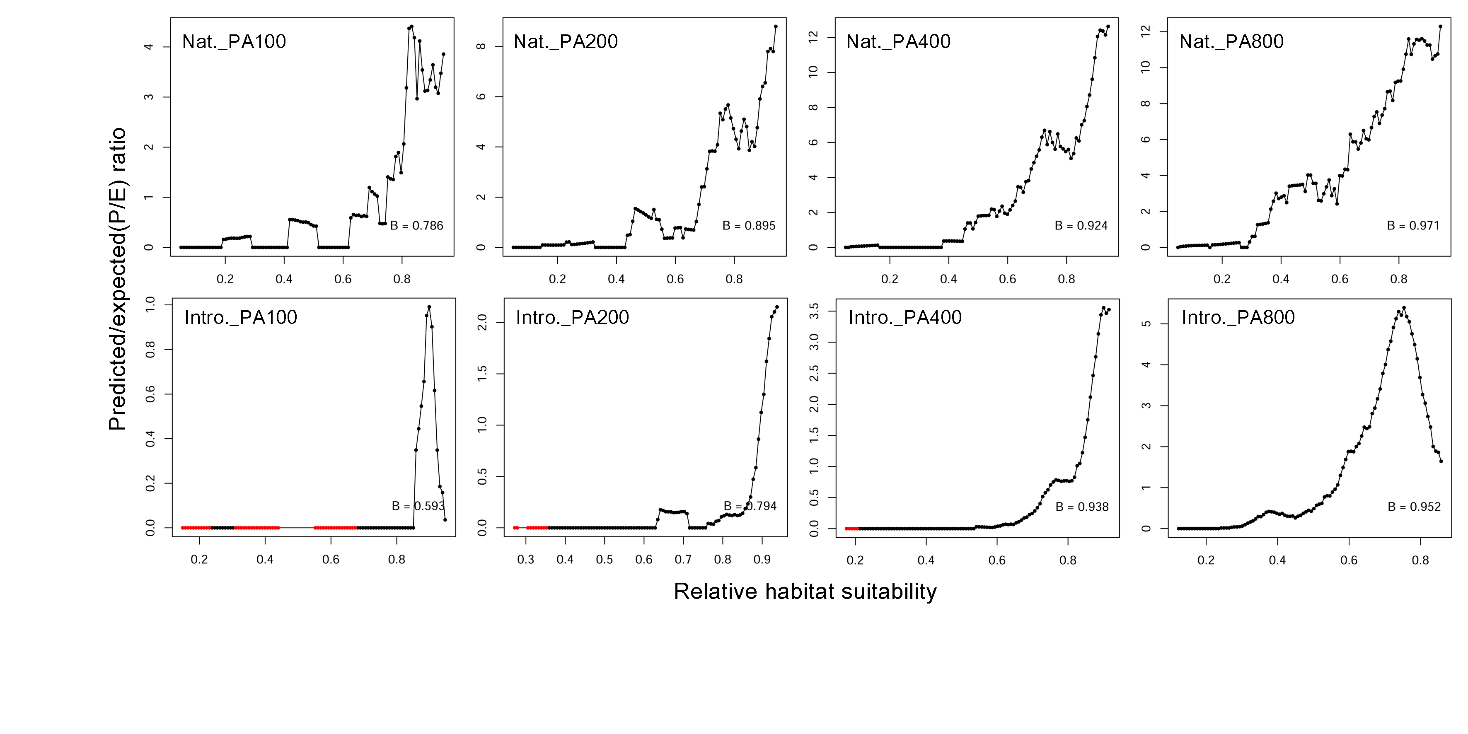


**S1 Fig. Predicted versus expected (P/E) curve and continuous Boyce index (CBI) based on the Spearman rank analysis for suitable habitat map evaluation depending on the number of pseudo-absences (PS).** The upper boxplots represent native habitat (Nat.) and the lower boxplots represent introduced habitat (Int.).
