# Supplementary figures and images for: Predicting the spatio-temporal distribution of the invasive alien plant Andropogon virginicus, in the South Korean peninsula considering long-distance dispersal capacities
Source: PLoS One. 2023 Nov 14;18(11):e0291365. doi: 10.1371/journal.pone.0291365 (PMC10645320; doi:10.1371/journal.pone.0291365)

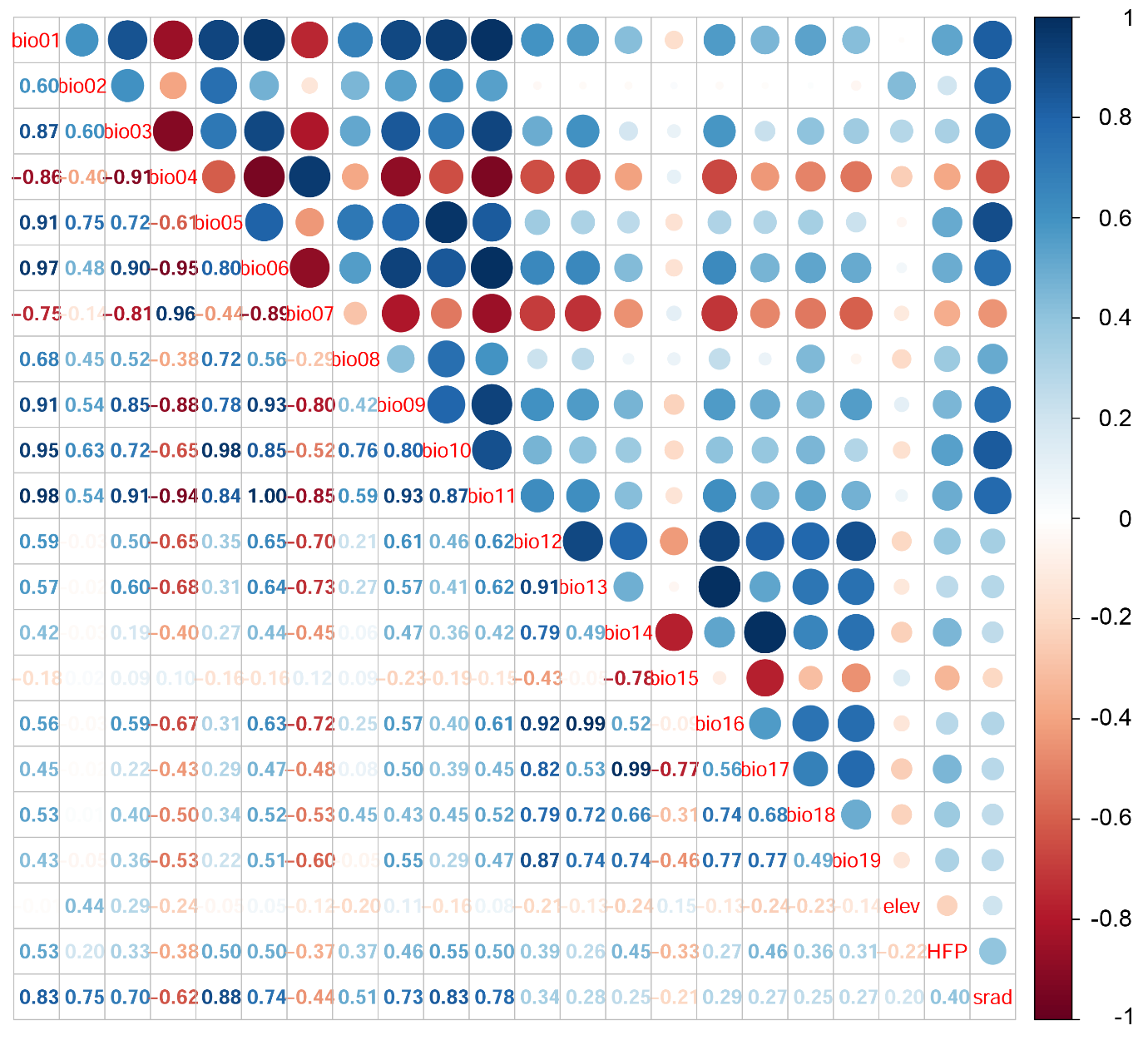


**S2 Fig. Spearman correlation matrix for environmental variables in North America.**

Supplement: S2 Fig — (DOCX) [file pone.0291365.s002.docx]
