# Supplementary material for: Predicting the spatio-temporal distribution of the invasive alien plant Andropogon virginicus, in the South Korean peninsula considering long-distance dispersal capacities
Source: PLoS One. 2023 Nov 14;18(11):e0291365. doi: 10.1371/journal.pone.0291365 (PMC10645320; doi:10.1371/journal.pone.0291365)
Supplement: S3 Fig — (A) The suitable habitat across its native distribution in North America in 2020. Predicted habitat suitability in the introduced regions in 2021 (B), 2041 (C), and 2061 (D). (DOCX) [file pone.0291365.s003.docx]

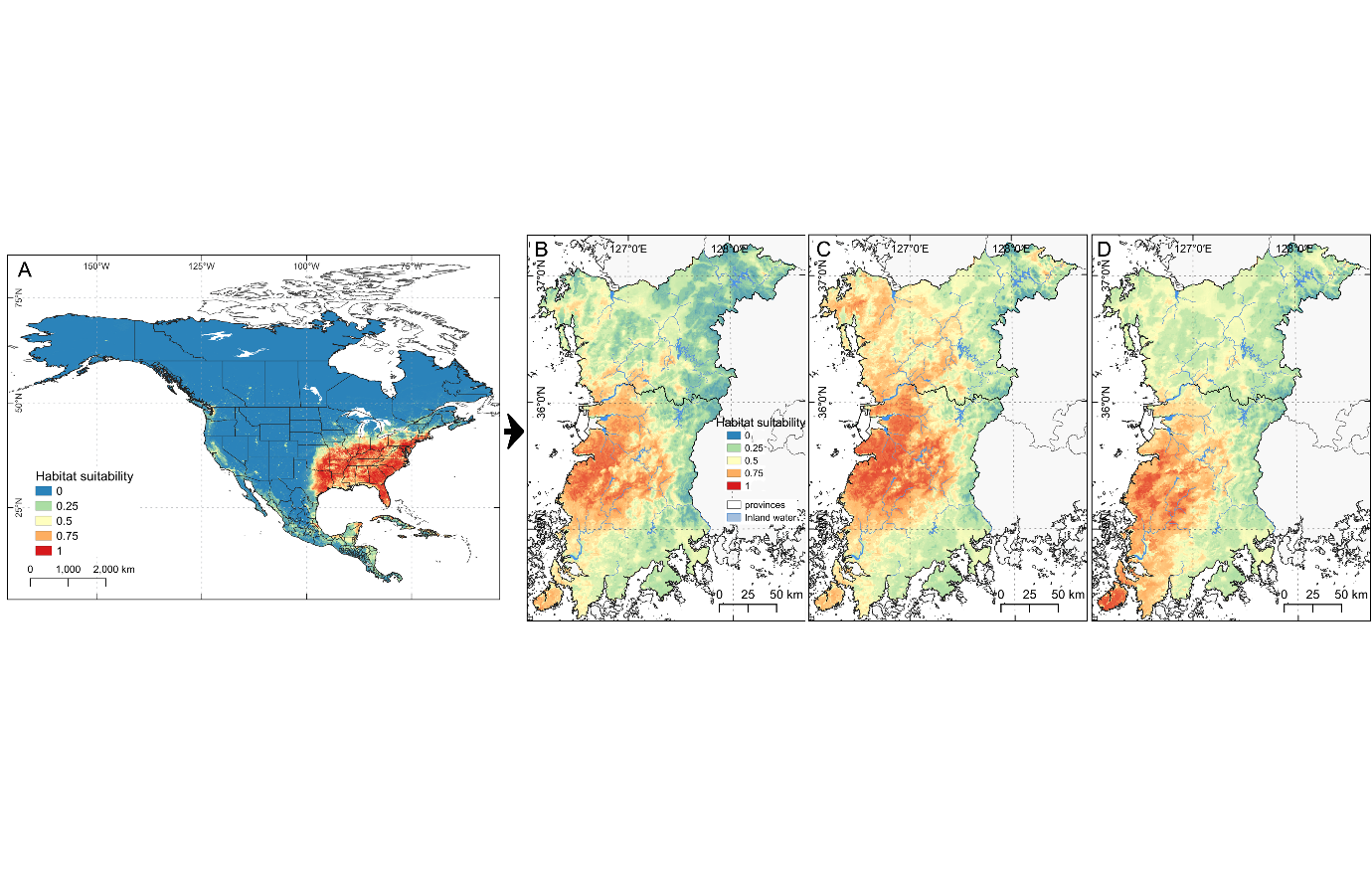


**S3 Fig. Maps of the suitable habitat for *A. virginicus.*** (A) The suitable habitat across its native distribution in North America in 2020. Predicted habitat suitability in the introduced regions in 2021 (B), 2041 (C), and 2061 (D).
