# Supplementary material for: Predicting the spatio-temporal distribution of the invasive alien plant Andropogon virginicus, in the South Korean peninsula considering long-distance dispersal capacities
Source: PLoS One. 2023 Nov 14;18(11):e0291365. doi: 10.1371/journal.pone.0291365 (PMC10645320; doi:10.1371/journal.pone.0291365)
Supplement: S4 Fig — [(A) F0.1_10km, (B) F0.05_10km, (C) F0.01_10km, (D) F0.1_30km, (E) F0.05_30km, (F) F0.01_30km)]. Two blue dots indicate the regions detected in 2009 and red dots indicate the regions detected in 2021 and 2022. (DOCX) [file pone.0291365.s004.docx]

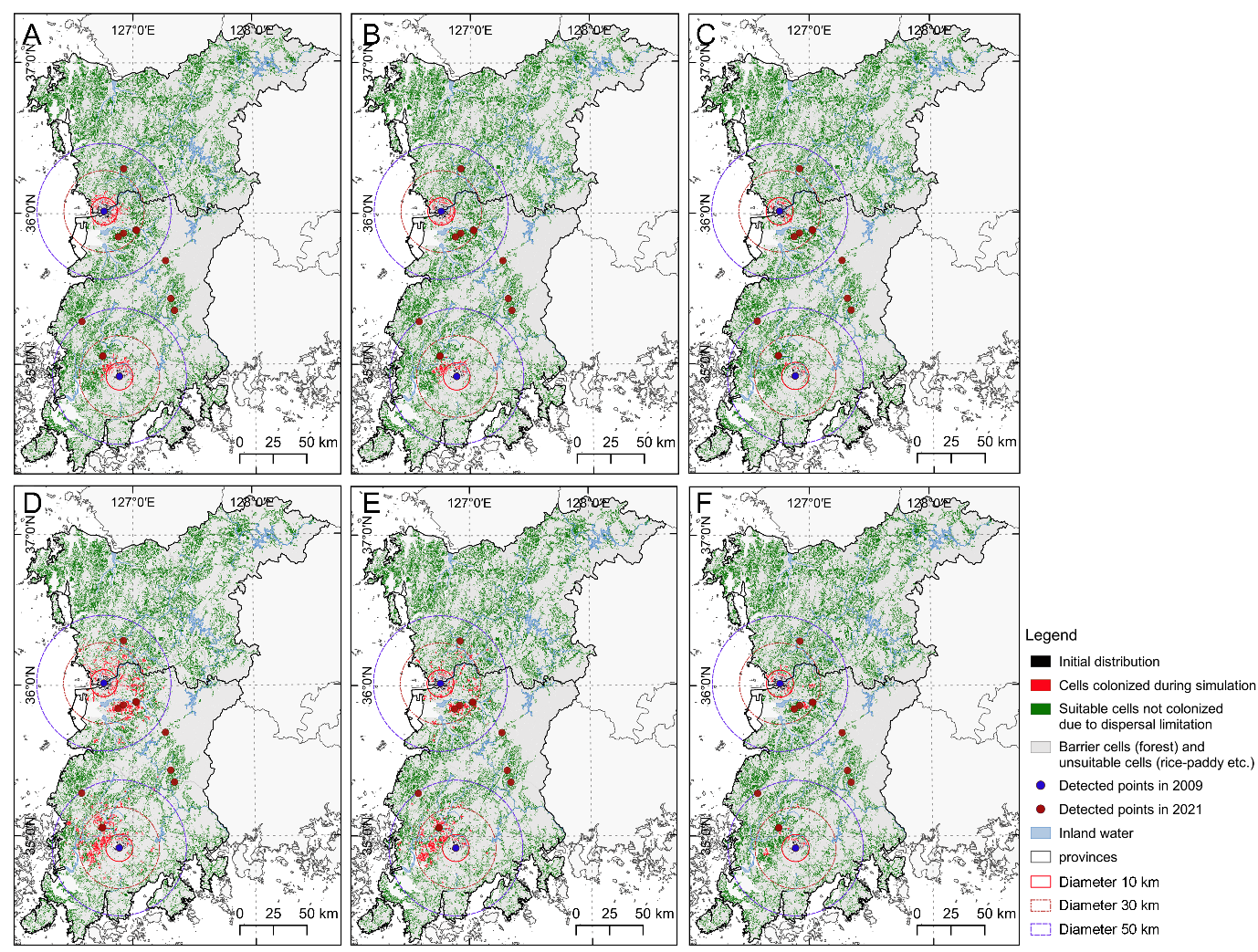


**S4 Fig. Estimated spatial distribution of *A. virginicus* from 2009 to 2021 under six LDD parameter sets.** [(A) F0.1_10km, (B) F0.05_10km, (C) F0.01_10km, (D) F0.1_30km, (E) F0.05_30km, (F) F0.01_30km)]. Two blue dots indicate the regions detected in 2009 and red dots indicate the regions detected in 2021 and 2022.
