# Supplementary material for: Predicting the spatio-temporal distribution of the invasive alien plant Andropogon virginicus, in the South Korean peninsula considering long-distance dispersal capacities
Source: PLoS One. 2023 Nov 14;18(11):e0291365. doi: 10.1371/journal.pone.0291365 (PMC10645320; doi:10.1371/journal.pone.0291365)
Supplement: S1 Table — CBI: continuous Boyce index, AUC: area under the receiver operating characteristic curve, Kappa statistic, TSS: true skill statistic. (DOCX) [file pone.0291365.s005.docx]

**S1 Table. Model validation statistics (CBI, AUC, Kappa, and TSS) for habitat suitability models depending on the number of pseudo-absence points.** CBI: continuous Boyce index, AUC: area under the receiver operating characteristic curve, Kappa: Kappa statistic, TSS: true skill statistic

| The number of Pseudo-absence points | North America  (Native habitat) | | | | South Korea  (Introduced habitat) | | | |
| --- | --- | --- | --- | --- | --- | --- | --- | --- |
|  | CBI | AUC | Kappa | TSS | CBI | AUC | Kappa | TSS |
| 100 | 0.786 | 0.946 | 0.877 | 0.872 | 0.593 | 0.815 | 0.047 | 0.062 |
| 200 | 0.895 | 0.990 | 0.883 | 0.883 | 0.794 | 0.869 | 0.541 | 0.635 |
| 400 | 0.924 | 0.990 | 0.933 | 0.949 | 0.938 | 0.948 | 0.773 | 0.830 |
| 800 | **0.971** | **0.992** | **0.912** | **0.916** | **0.952** | **0.956** | **0.838** | **0.838** |
